# Mental Health and Wellbeing at Schools: Health Promotion in Primary Schools with the Use of Digital Methods

**DOI:** 10.3390/children8050345

**Published:** 2021-04-27

**Authors:** Evanthia Sakellari, Venetia Notara, Areti Lagiou, Natalja Fatkulina, Svetla Ivanova, Joonas Korhonen, Nevenka Kregar Velikonja, Valentina Lalova, Camilla Laaksonen, Gergana Petrova, Mari Lahti

**Affiliations:** 1 Department of Public and Community Health, School of Public Health, University of West Attica, 12243 Athens, Greece; sakellari@uniwa.gr (E.S.); vnotara@uniwa.gr (V.N.); alagiou@uniwa.gr (A.L.); 2 Laboratory of Hygiene and Epidemiology, School of Public Health, University of West Attica, 12243 Athens, Greece; 3 Department of Nursing, Institute of Health Sciences, Faculty of Medicine, Vilnius University, 01513 Vilnius, Lithuania; natalja.fatkulina@mf.vu.lt; 4 Department of Nursing, Faculty of Public Health, Medical University of Plovdiv, 4002 Plovdiv, Bulgaria; svetla.ivanova@mu-plovdiv.bg (S.I.); Valentina.Lalova@mu-plovdiv.bg (V.L.); Gergana.Petrova@mu-plovdiv.bg (G.P.); 5 Faculty of Health and Well-Being, Turku University of Applied Sciences, 20520 Turku, Finland; joonas.korhonen@turkuamk.fi (J.K.); camilla.laaksonen@turkuamk.fi (C.L.); 6 Faculty of Health Sciences, University of Novo Mesto, 8000 Novo Mesto, Slovenia; nevenka.kregar-velikonja@uni-nm.si; 7 Department of Nursing Science, Faculty of Medicine, University of Turku, 20014 Turku, Finland

**Keywords:** mental health, wellbeing, digital methods, health promotion interventions, primary school children, Well@School

## Abstract

Mental health disorders among primary school children remain a crucial issue. Early health promotion interventions can positively affect and prevent the onset of mental disorders. Promising digital mental health methods have been implemented for adolescents and youths with scarce evidence among younger ages. Therefore, the aim of the current systematic review was to identify health promotion interventions on mental health and wellbeing, with the use of digital methods, delivered in primary school settings. Six digital interventions have been identified, three of which were targeting teachers and the others students. Regardless of the limited number of studies, the effectiveness of the web-based interventions upon teachers’ knowledge and attitudes and the positive impact on children’s behavioral improvements has been documented. The lack of adequate evidence highlights the need for further research in the field. The current review provides information for professionals working in primary schools useful for the design and implementation of effective mental health and wellbeing interventions.

## 1. Introduction

Psychiatric disorders are the leading cause of disability in young people worldwide [1] and according to previous research [2,3], less than 20% of children in need of mental health services receive them. According to the OECD, mental disorders account for one of the largest and fastest growing categories of disease burden worldwide [4]. Approximately half of all psychiatric disorders originate by midadolescence and up to 20% of children and adolescents worldwide will experience some type of psychiatric disorder [5,6,7]. A global burden of disease study in 2010 revealed that mental and substance use disorders account for the highest proportion of DALYs in the age group 10–29 years and reported a 37.6% increase in these disorders between 1990 and 2010 [8]. Similarly, a recent report of the World Health Organization (WHO) highlighted the prevalence of mental ill health, i.e., depression, among children and adolescents below the age of 15 years [9]. In the UK, almost 10% of primary school children are faced with at least one mental disorder, while 3.4% suffer from two or more disorders, with the most prevalent being behavioral and emotional disorders [10]. It is documented that by the age of 14, mental illnesses have already arisen that will persist during adulthood [11,12]. It is therefore important to prevent the onset of mental ill health in the early stages of life, due to the fact that during childhood social and emotional skills are developed and can be negatively influenced by several risk factors [13].

Under this spectrum, schools have been reported to provide ideal settings for health promotion and well-implemented school-based interventions that can promote the physical and mental health of school children [14,15,16]. School-based programs can reach practically all groups of children and parents, since more than 90% of school aged (age 5–15) children attend primary schools globally [17]. Common areas of school-based health promotion interventions are the encouragement of physical activity and fitness [18,19], nutrition [20], prevent substances’ use [21,22] and mental health promotion [23]. Nevertheless, integrating mental health related education on school curriculums through training of teachers has been shown as an effective and sustainable way to increase recognition of health problems, knowledge, positive help-seeking efficacy and decrease stigma [24].

Despite the availability of school-based mental health promotion interventions, they do not achieve the desired effect. To increase the effect of traditional methods, new promising online methods for mental health promotion have been developed and implemented with a number of studies in secondary schools [25,26,27,28,29,30,31]. The rapid growth in the use of online technologies among youth provides an opportunity to increase access to evidence-based mental health resources [32]. The literature emphasizes the academic benefits of mental health promotion in schools and there is strong evidence for the effectiveness of interventions to support positive gains in students’ social-emotional and academic outcomes [33,34]. In 2013, the WHO stated that by combined educational interventions, health literate and healthier, families and communities gain success and academic prosperity in later life [35].

The aim of the current systematic review was to identify health promotion interventions on mental health and wellbeing, with the use of digital methods, in primary school settings and provide useful information for those who design and implement health promotion activities in primary schools. The ultimate goal of this review is to summarize up-to date findings in this area and encourage all interested stakeholders to implement new approaches of mental health and wellbeing interventions within schools.

The research questions which guided the current systematic review are the following: 

What are the health promotion interventions on mental health and wellbeing in primary schools using digital methods?

What are the specific aims of these interventions?

What types of digital methods have been used in these interventions?

Which are the tools used to evaluate the impact of these interventions?

What are the outcomes of these interventions?

## 2. Materials and Methods

The current paper is a part of the Erasmus+ Strategic Partnership project Well@School (more information at https://wellatschool.turkuamk.fi/ (accessed on 2 February 2020), whose aim is to strengthen the education of higher education institutions by modernizing digital mental health promotion in schools. All the existing data in the field of use of digital methods for implementation of mental health and wellbeing interventions in primary school settings were accumulated. The guidelines set by Glasziou et al. were used to conduct this systematic review [36]. Evidence-based studies, with diverse methodologies, were explored and research questions and a search strategy were formulated based on the inclusion/exclusion criteria. Subsequently, data analyses as well as presentation of the final results were reported.

### 2.1. Inclusion and Exclusion Criteria

All the inclusion and exclusion criteria were applied to the retrieved articles. No timeframe was specified. The criteria are presented in Table 1.

### 2.2. Literature Search

A literature search was conducted by the research team during October 2020–November 2020 using the databases of PubMed/Medline, Cochrane, PsycINFO-ProQuest Research Library, Scopus, Web of Science, CINAHL and Google Scholar (limited to the last five years, for grey literature: dissertations, organizational reports, etc.). The search strategy was based on the following terms: (digital methods *OR* digital tools *OR* applications *OR* technology *OR* computer *OR* ICT) AND (mental health *OR* mental illness *OR* mental condition *OR* mental state *OR* psychological health) AND (wellbeing *OR* wellbeing *OR* wellness *OR* healthy *OR* welfare *OR* happiness) AND (health promotion interventions *OR* health promotion strategies *OR* health promotion approaches *OR* health promotion programs/programmes *OR* health promotion projects *OR* health promotion activities) AND (primary school children *OR* primary school setting/s *OR* primary school education)**.** The relevant literature was searched in December 2020, with no restriction regarding publication dates.

**Figure 1:**
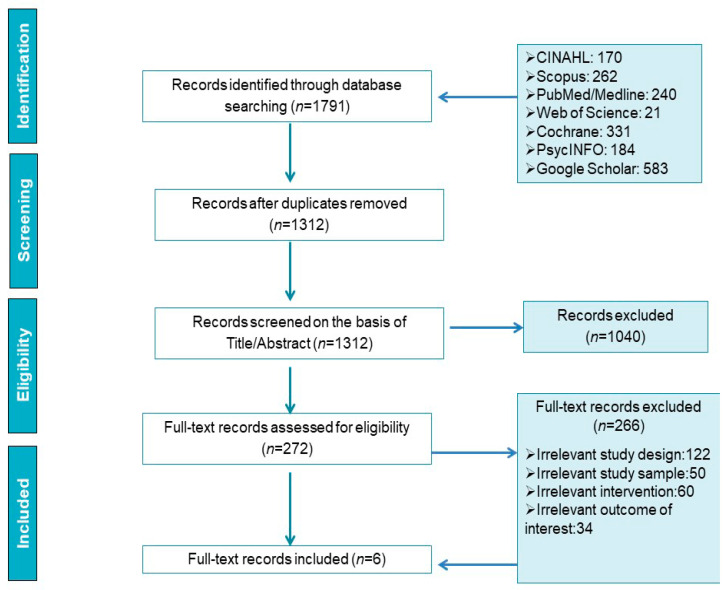
Flow diagram of the search process.

The search process, followed the PRISMA guidelines [37], is presented in Figure 1. Initially, 1791 articles were retrieved. After duplicate removal, 1312 articles remained for title and abstract screening. The 1040 articles were removed on the basis of the title and abstract since they were not relevant to the aim of the present review. Among the rest 272 articles, 122 had no digital intervention, 50 had an irrelevant study sample (i.e., mainly focused on high school students and youths), 60 had irrelevant interventions and were focused on digital intervention regarding literacy and educational skills and in 34 the full text was written in German or French or they were commentaries and meta-analyses. Therefore, six studies met the inclusion criteria and were assessed in the final review.

### 2.3. Data Analysis

The eligibility of the papers was assessed with a hierarchical structure starting from the title and abstract, which was carried out by Well@School team, and subsequently the eligible articles were screened in full text, which was carried out by two team members. Data extraction was firstly undertaken by one author (VN); afterwards, it was cross checked by a second one (ES) and a total agreement was achieved. The extraction included author name/publication year, country of study, study aim, participants, study design, content and duration of intervention, assessment tools/testing time and main findings.

## 3. Results

Six studies met the inclusion criteria and were further assessed. These studies were conducted in different countries worldwide, two in the USA, one in Canada, one in Brazil, one in the UK, and one in Hong Kong, and they were recently published—i.e., after 2012. The studies used experimental designs with the number of participants ranging from 12 to 18,896 (Table 2).

### 3.1. Aims of the Studies

The aim of the selected studies (Table 2) was to explore the effectiveness of digital mental health interventions in primary school settings, targeting either teachers or children. The studies conducted among teachers were mainly focused on skills of teachers to influence students’ knowledge, attitudes and behaviors. More specifically, one study, conducted in the USA, assessed elementary school teachers’ ability to influence attitudes and behaviors of students with psychological distress using an online mental health role-play simulation [38]; the other study, conducted in Brazil, applied web-based educational programs for teachers to gain knowledge and modify attitudes and beliefs about students with mental disorders [39]; the study in Canada applied web-based program for teachers to improve knowledge and increase their ability to reduce symptoms and impairment of students with attention-deficit/hyperactivity disorder (ADHD) in a school environment [40].

The studies that were conducted among students were focused on the improvement of their mental wellbeing. More specifically, the study conducted in Hong Kong aimed to enhance social and communication skills, problem-solving skills, empathy, and emotional competence of students [41]. Computerized cognitive behavior therapy (cCBT), that aimed at identifying primary school children at high risk of anxiety and several behavioral problems as well as helping them to reduce such behaviors, was applied in the USA [42]. The other cCBT program, developed in the UK, was intended to be used as a supportive tool in mild emotional problems of anxiety and low mood rather than as a unique intervention in more severe problems [43].

### 3.2. Interventions

The interventions varied among the studies (Table 2), all of which were designed to address their aims and diverse digital (technological) tools were applied. In the studies on teachers, Pereira et al. conducted two simultaneous interventions—web-based interactive education (WBIE), text and video-based education (TVBE)—and a control group. One interesting finding was the fact that not only teachers in the WBIE group but also the control group showed increased levels of knowledge compared to those in the TVBE group [39]. Long et al. used an online mental health role-play simulation tool, the “At-Risk for Elementary School Educators”. A set of virtual dialogues (conversations) between teachers and students or parents was provided and the teachers were able to see the respondents’ reactions so as to select a less critical and judgmental conversation [38]. Barnett et al. used a variety of technological tools for the online intervention (i.e., discussion board, intrasystem email, assessments and web links) [40].

In the studies for students, the digital game-based program, “The Adventures of DoReMiFa”, was combined with school-based teaching and incorporated storylines, dialogues, problem-solving, mini-games, etc. [41]. Two computerized cognitive behavior therapy (cCBT) programs were used—i.e., the “Camp Cope-A-Lot” (CCAL) [42] and the ‘Think, Feel, Do” [43]. The CCAL was a 4-week computer-assisted intervention program which included skill-building activities and anxiety coping strategies. “Think, Feel, Do” is an interactive, multimedia CD-ROM which involved quizzes, video clips, music and animation cartoons guiding users through various activities.

### 3.3. Evaluation Tools

For the assessment process, self-reports and validated scales were administered before and after the completion of the programs (Table 2). The scales were used for measuring student’s depression, self-esteem and negative thinking (i.e., the Spence Children’s Anxiety Scale Child and Parent Version, Adolescent Well-Being Scale, Schema Questionnaire for Children and Rosenberg Self Esteem Inventory) [43] or fears/worries and behavioral weaknesses (i.e., the Beck Anxiety Inventory for Youth, BASC-3 Behavioral and Emotional Screening System, Children’s Usage Rating Profile and the Usage Rating Profile–Intervention Revised) [42]. Validated scales were also used for measuring teacher’s attitudes and behavioral changes (i.e., The Gatekeeper Behavior Scale) [38] or anxiety disorders, mental health knowledge, negative self-statements, etc., in children (i.e., The Screen for Child Anxiety—Related Emotional Disorder, Mental Health Knowledge Checklist, Children’s Automatic Thoughts Scale-Negative or Positive, Interpersonal Reactivity Index, Rosenberg Self-Esteem Scale) [41]. Questionnaires were also used for assessing knowledge, beliefs, and attitudes. Specifically, the assessment tool used by Pereira et al. consisted of 48 questions, of which 27 assessed teachers’ knowledge (with dichotomous responses) and 21 assessed beliefs and attitudes on child mental health. The 21 questions were based on a questionnaire from the World Psychiatric Association Taskforce on Awareness [39]. Knowledge, attitudes, and behavior were measured before and after intervention through teachers’ self-reporting, which authors regarded as a limitation [40].

Finally, all studies evaluated the effectiveness of the interventions within a time range from immediately after the intervention up to six months.

### 3.4. Intervention Outcomes

The outcomes of these interventions were varied but somehow uniform (Table 2). In Pereira et al.’s study, no effect on teacher’s attitudes with regard to mental health was observed. The possible reasons for this are either due to the difficulty in measuring modifications or to the intervention itself or the low participation adherence and motivation in a certain program. However, it was found that, for knowledge, the web-based interactive tools were more effective and acceptable than the traditional educational tools [39]. Long et al.’s study, regardless of the limited follow-up time, helped teachers to react properly to students experiencing psychological distress [38]. Even though no changes in teaching behaviors were reported, the results regarding teachers’ increased knowledge and attitudes towards ADHD were rather positive and encouraging. Larger online trial to evaluate the effectiveness of the certain web-based intervention was suggested as they were regarded an effective tool in the improvement of teachers’ knowledge and attitudes [40]. The digital game-based program “The Adventures of DoReMiFa” was found to be helpful for students to increase several skills such as problem-solving, communication and cognitive skills, emotional competence, empathy, etc. The authors suggested that, regardless of the limitations of the intervention, preventive mental health promotion programs should be implemented in the early stages of school life so as to increase their effectiveness in mental wellbeing [41]. The cCBT program, “Camp Cope-A-Lot” (CCAL), was rather effective in reducing student’s internalizing behaviors (i.e., anxiety and depression) [42]. In the same line, the results of the cCBT program “Think, Feel, Do”, despite its limitations (i.e., small sample size, restricted follow-up), showed that cCBT can have a positive impact in terms of reducing depression and anxiety in high-risk students [43].

## 4. Discussion

There is evidence of the association between mental health problems during childhood and psychiatric disorders through youth and adulthood [44]. Despite this, overall, research on mental health and wellbeing interventions using digital methods in primary school settings is limited worldwide, with only six studies satisfying the inclusion criteria of the current study. In addition, a recent systematic review of school-based universal mental health interventions, addressing the common types of mental health disorders among primary school children, identified a small number (nine) of published studies [45]. A review on codesign of digital mental health technologies with children and young people identified 30 digital mental health technologies, but the majority (25/30) of them targeted young populations; among the five studies also targeting the child populations, three were referred by personal communication [46].

As previously mentioned, the current review paper identified six digital interventions in primary school settings having either teachers [38,39,40] or students as target groups [41,42,43]. One of them was referred to elementary school teachers for the management of children with ADHD [40]. Despite the limitations, such as the small sample size and the lack of a control group, the study documented the effectiveness of the web-based intervention upon teachers’ knowledge and attitudes regarding ADHD. No changes in teaching behaviors were observed in that study, probably due to technological limitations. It is documented that opportunities for prevention and early identification of mental health problems are often missed in youth as a consequence of the overburdening of the mental health care system [47]. In a systematic and meta-review study exploring the promise and potential of digital health interventions for several mental health disorders, it was concluded that these interventions could not be recommended for the treatment of ADHD [48]. A recent randomized control trial of digital intervention in children with ADHD observed improvements of inattentive symptoms and, in line with our review results, the authors concluded that future research is needed for more robust results [49].

The lack of digital mental health interventions and the expressed need for the effective management of children with mental disorders in primary school settings has led to the implementation of two web-based programs for primary school teachers [38,39]. In both programs, the aftermath evaluation process showed increased levels of knowledge regarding mental health; however, no such improvements were observed in teachers’ beliefs and attitudes [39]. A suggested explanation was the poor adherence and low motivation to participate in the program. Role-play simulations helped teachers to correct mistakes when confronting students with psychological distress [38]. However, the short time of follow-up did not confirm the long-term impact on teachers’ behavior.

Given the fact that children are familiar with new technologies, digital-based treatments could prove beneficial for mental health promotion, with more controlled clinical trials confirming their effectiveness [50]. Digital-based technologies for managing mental health difficulties in children and young people are more specifically focused on anxiety, depression, suicidal thoughts, self-harm, substance misuse, crises and emotional health [46]. Two of the digital interventions on primary school students with anxiety disorders provided positive results not only in the identification of high-risk students for anxiety and other behavioral problems [42], but also among children with emotional problems [43]. Even though both studies had several limitations, it was concluded that computerized cognitive behavioral therapy (cCBT) programs provide a costless way of addressing adverse behavioral profiles. Similarly, internet CBT was associated with significant improvements in anxiety postintervention and at follow-up compared to the waitlist [51]. Positive feedback on digital health interventions has been received in previous studies—for example, child satisfaction receiving computer-assisted CBT was high [52]. A recent meta-analysis to assess the effectiveness of cCBT for anxiety and depression in youths concluded that such interventions could be promising treatments in children and adolescents [53]. In the same context, another meta-analysis indicated that internet-based interventions may be effective in anxiety symptoms but not in depression [54]. The results of a systematic review of online mental health promotion and prevention interventions among youth aged 12–25 years showed a significant positive effect of computerized cognitive behavioral therapy on adolescents’ and emerging adults’ anxiety and depression symptoms [32]. Similarly, a study among junior high schools showed that a universal, internet-based approach to stress management is practical and feasible and likely to prove effective [55].

The digital game-based intervention showed that if used in combination with school-based teaching, the impact would be greater. Specifically, the classroom teaching involved several activities (i.e., role-play and card games) relevant to the digital game so as to increase students’ emotional competence, communication skills, problem-solving, empathy, etc. [41]. The authors evaluated the intervention program 6 months later and they observed significant improvements in mental health knowledge but not in anxiety symptoms, negative thoughts and self-esteem. It is documented that promoting children’s health with active digital games seems quite promising even among other fields of health, such as physical activity [56]. Self-management behaviors of young people with chronic diseases [57] or support of pediatric cancer patients, where a statistically significant decrease in general health problems and a trend toward a decrease in depression and anxiety symptoms were observed after implementation of a mobile health game [58].

The current review paper found a small number of digital mental health interventions for children in primary school settings. Likewise, in a recent systematic review, even though the majority of the identified studies reported interventions with the use of the internet, the authors concluded that universal and selective primary prevention interventions were also not available to children aged ≤9 years [59].

The six studies included in the current review were carried out in different social, political and cultural contexts. However, conclusions about the roles that these differences play in mental health problems among young persons cannot be made.

### Limitations

This systematic review revealed only six studies that were conducted in primary school settings aiming to promote mental health and wellbeing using digital methodology. If the review was broader, including secondary and tertiary education, more studies would have been found. Furthermore, since the identified studies included a wide range of different methodological approaches used in the interventions, a reliable representation of the most effective method is difficult to provide. However, useful insights are provided for the mental health and wellbeing activities taking place in primary school settings. Finally, the current review was restricted to publications in the English language and thus there might be more studies on the targeted topic that are not included here.

## 5. Conclusions

Digital mental health interventions in primary school settings are scarce. The limited number of identified studies showed promising results. Despite the lack of adequate evidence, there is unequivocal need for further studies in the field of mental health with digital methods, especially among primary school children. Technology is regarded as an important and cost-effective tool in the management of mental disorders in school settings, where children spend most of their daytime. Finally, professionals working in primary schools can utilize the information provided in the current literature and design and implement effective mental health and wellbeing interventions.

## Figures and Tables

**Table 1:** Inclusion and exclusion criteria.

**Inclusion Criteria**
1. Articles written in English language
2. Empirical studies
3. Dissertations and/or case reports
4. Organizational reports and guidelines
5. Intervention studies
6. Conducted in primary school settings
7. Participants were any staff members and/or students of primary schools
**Exclusion Criteria**
1. Systematic and meta-analyses articles
2. Not intervention studies
3. Did not use digital mental health interventions
4. Editorials and/or commentaries
5. Book reviews and/or letters
6. Articles not focused on mental health and wellbeing of primary school children
7. Study protocols

**Table 2:** Review of final six studies.

Author/Year	Country	Study Aim	Number of Participants	Content of Intervention	Duration of Intervention	Evaluation Tools/Test Timing	Intervention Outcomes
Sanders et al., 2019	USA	To address the feasibility and effectiveness of an abbreviated cCBT software program, “Camp Cope-A-Lot” (CCAL), for elementary students at risk for anxiety and other behavioral problems.	26 students (7–11 years) -Treatment group (*n =* 11) -Control group (*n =* 15)	The abbreviated cCBT software program, Camp Cope-A-Lot (CCAL)	6 sessions lasting 20–30 min for 4 weeks	-Beck Anxiety Inventory for Youth (BAI-Y) -BASC—Behavioral -Emotional Screening System (BASC-3 BESS -Children’s Usage Rating Profile (CURP) -Usage Rating Profile–Intervention Revised (URP-IR) All administered at the end of the CCAL program.	Successful impact to ease the internalizing symptoms of students in an after-school setting.
Shum et al., 2019	Hong Kong—China	To examine the effectiveness of a school-based digital game-based intervention program “DoReMiFa” with the combination of a CBT and positive psychology model.	459 children (8 to 12 years) -Intervention group (*n =* 264) -Control group (*n =* 195)	-The Adventures of DoReMiFa, a digital game–based lesson -School-based lesson	11 digital game-based lessons lasting around 25 to 60 min/lesson	-The Screen for Child Anxiety-Related Emotional Disorders -Mental Health Knowledge Checklist -Children’s Automatic Thoughts Scale-Negative or Positive -Interpersonal Reactivity Index -Rosenberg Self-Esteem Scale Administered at: -preintervention stage -2 weeks after completion of -6-month follow-up	Effective results in the mental health knowledge even 6 months after the intervention.
Long et al., 2018	U.S.A.	To evaluate the impact of the “At-Risk for Elementary School Educators” online mental health role-play simulation for elementary school teachers to confront students with psychological distress.	18,896 elementary school teachers (mean age 41 years). -Intervention group (*n =* 9427) -Control group (*n =* 9469).	At-Risk for Elementary School Educators, a self-paced online simulation.	A 45 to 90 min online role-play simulation	The Gatekeeper Behavior Scale: -at baseline -post-test -3-month follow-up	Effective results in teacher’s preparedness, likelihood, and self-efficacy to perform positive gatekeeping behaviors for students with psychological distress.
Pereira and Wen, 2015	Brazil	To develop a web-based program, to educate primary school teachers on child mental disorders and to test its effectiveness compared with other methods delivered or with no intervention.	115 teachers (mean age 40.3) -Web-based interactive education (WBIE) group (*n =* 52) -Text- and video-based education (TVBE) group (*n =* 32) -Waiting list (WL, no intervention) (*n =* 31).	-Educational videos -Website tutorial -Internet discussion forum -Web conference -Written support text	9 h of training (three-hour session/week for 3 weeks)	Questionnaires assessing knowledge, beliefs and attitudes: -at the preintervention stage- immediately after the intervention	-Τhe WBIE group showed greater gains in knowledge than other groups. -The WL group gained more knowledge than did those trained with the text- and video-based program.
Attwood et al., 2012	UK	To evaluate the computerized CBT program “Think, Feel, Do” in two studies: Study 1: a universal study Study 2: targeted at children with mild/ moderate emotional problems of anxiety or low mood.	-Study 1: 13 boys (10 and 12 years). -Study 2: 12 children (9 boys and 3 girls) (10 to 16 yr)	-Study 1: Boys were assigned to either cCBT or a matched computer time (gaming) condition. -Study 2: The cCBT was delivered by the school nurse. “Think, Feel, Do” consisted of quizzes, practical exercises, video clips, music and animation was delivered. Online games were also used.	6 sessions lasting 45 min/week or every 2 weeks, during class time.	-Spence Children’s Anxiety Scale Child and Parent Version (SCAS) -Adolescent Well-Being Scale (AWS) -Rosenberg Self Esteem Inventory (RSEI) -Schema Questionnaire for Children (SQC) Assessments were completed within two weeks after the completion of the intervention	The cCBT “Think, Feel, Do” can have positive effects on anxiety and low mood symptoms
Barnett et al., 2012	Canada	To determine whether a web-based medium is an effective tool for supporting knowledge, attitude, and behavior changes in teachers of elementary school children with ADHD.	19 elementary school teachers (mean age 36.9 years)	-Discussion Board -Intrasystem email -Web links	7 sessions: 1 session/week	Self-report measures after the intervention	Increased knowledge on managing ADHD in the classroom was documented.

## Data Availability

No new data were created or analyzed in this study. Data sharing is not applicable to this article.
